# Miliary Tuberculosis With Immune Thrombocytopenia in 50‐Year‐Old Ethiopian Woman: A Case Report and Brief Review of Literature

**DOI:** 10.1002/ccr3.71922

**Published:** 2026-01-26

**Authors:** Hayatu Awel Abdela, Tamirat Godebo Woyimo, Ragasa Getachew Bayisa, Sisay Tagese Tafese

**Affiliations:** ^1^ Department of Internal Medicine, School of Medicine, College of Medicine and Health Sciences Wolkite University Wolkite Ethiopia; ^2^ Department of Internal Medicine, School of Medicine, College of Medicine and Health Sciences Jimma University Jimma Ethiopia

**Keywords:** anti‐tuberculosis therapy, autoimmune diseases, Ethiopia, hematology, immune thrombocytopenia, miliary tuberculosis, severe thrombocytopenia

## Abstract

Immune thrombocytopenia (ITP) is a rare but recognized hematologic complication of tuberculosis (TB), particularly miliary TB. We present a case of a 50‐year‐old female with severe thrombocytopenia and hemorrhagic manifestations secondary to miliary TB. The patient presented with mucocutaneous bleeding, anemia, and constitutional symptoms. Investigations revealed severe thrombocytopenia (11,000/μL), erythroid hyperplasia on bone marrow aspiration, and radiographic findings consistent with miliary TB, later confirmed by sputum GeneXpert. Management included short‐course high‐dose dexamethasone for acute bleeding, followed by anti‐tuberculosis therapy (ATT), which led to sustained platelet recovery without further immunosuppression. This case highlights miliary TB as a reversible cause of secondary ITP and underscores the importance of investigating underlying infections in patients presenting with thrombocytopenia in TB‐endemic regions.

## Abstract (highlights)

**Key Clinical Message:**

In tuberculosis (TB)‐endemic regions, miliary tuberculosis should be considered a potential cause of severe immune thrombocytopenia. Early diagnosis and initiation of anti‐tuberculosis therapy can lead to complete hematologic recovery, obviating the need for prolonged immunosuppression.

**Learning Points:**

- TB should be considered in the differential diagnosis of secondary ITP, particularly in endemic areas and in patients with constitutional symptoms
- A brief course of corticosteroids may be appropriate for severe bleeding while awaiting ATT initiation
- ATT alone may be sufficient to achieve sustained platelet recovery in TB‐associated ITP.
- Bone marrow examination may not be necessary when a clear infectious etiology is identified.
- Long‐term follow‐up is essential to confirm durable remission.

## Graphical abstract

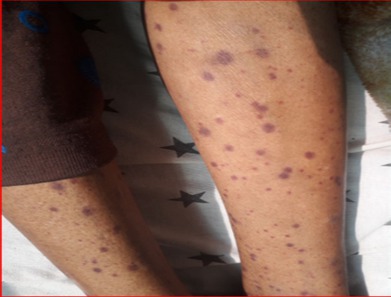

## 1 Introduction

Immune thrombocytopenia (ITP) is an acquired autoimmune bleeding disorder defined by isolated thrombocytopenia resulting from immune‐mediated platelet destruction, impaired platelet production, and platelet dysfunction as well [1, 2]. ITP occurs as a primary (idiopathic) disorder or secondarily in association with infections, autoimmune diseases, lymphoproliferative disorders, and certain medications; infections are a well‐recognized trigger of secondary ITP [3, 4].

Tuberculosis (TB)—particularly disseminated (miliary) forms—is an uncommon but increasingly recognized cause of secondary ITP. Although only a small number of well‐documented cases and case series have been published, reports consistently show that platelet counts may normalize after effective anti‐tuberculosis therapy (ATT), supporting a causal link in selected patients. The overall literature therefore characterizes TB‐associated ITP as rare but clinically important, especially in areas with high TB prevalence [5, 6].

Several mechanisms have been proposed to explain how TB can produce severe thrombocytopenia, including infection‐driven autoimmunity (antiplatelet antibodies and molecular mimicry), immune complex–mediated platelet destruction, impaired thrombopoiesis from bone‐marrow involvement or suppression, and increased platelet clearance related to systemic inflammation or splenic sequestration. The precise mechanism likely varies between patients and may combine several pathways [7, 8, 9]. Recognition of TB as a potential, reversible cause of ITP has important clinical implications in TB‐endemic settings such as Ethiopia, where the burden of TB remains substantial. Considering TB early in the diagnostic workup of otherwise unexplained severe thrombocytopenia can prompt timely microbiologic testing (e.g., GeneXpert or other rapid diagnostics) and initiation of ATT, which in many reported cases produced sustained hematologic recovery and avoided prolonged or unnecessary immunosuppression. Steroids, immunoglobulins (IVIG), anti‐D immunoglobulins were also used in the treatment of ITP associated with tuberculosis [10, 11]. Because corticosteroid therapy given in unrecognized active TB can worsen infection, identifying an infectious etiology before committing to long‐term immunosuppressive treatment is particularly relevant in these settings [12, 13].

We report a case of a 50‐year‐old Ethiopian woman who presented with severe thrombocytopenia and hemorrhagic manifestations that were ultimately attributed to miliary TB. This case adds to the limited literature on TB‐associated ITP and highlights the diagnostic and therapeutic considerations that clinicians should bear in mind in high‐burden regions.

## 2 Case History/Examination

A 50‐year‐old female with no prior chronic medical conditions presented with a 5‐day history of generalized petechial rash, gum bleeding, epistaxis, hematuria, and bloody diarrhea. She also reported chronic symptoms including productive cough, low‐grade fever, night sweats, loss of appetite, and weight loss.

**FIGURE 1:**
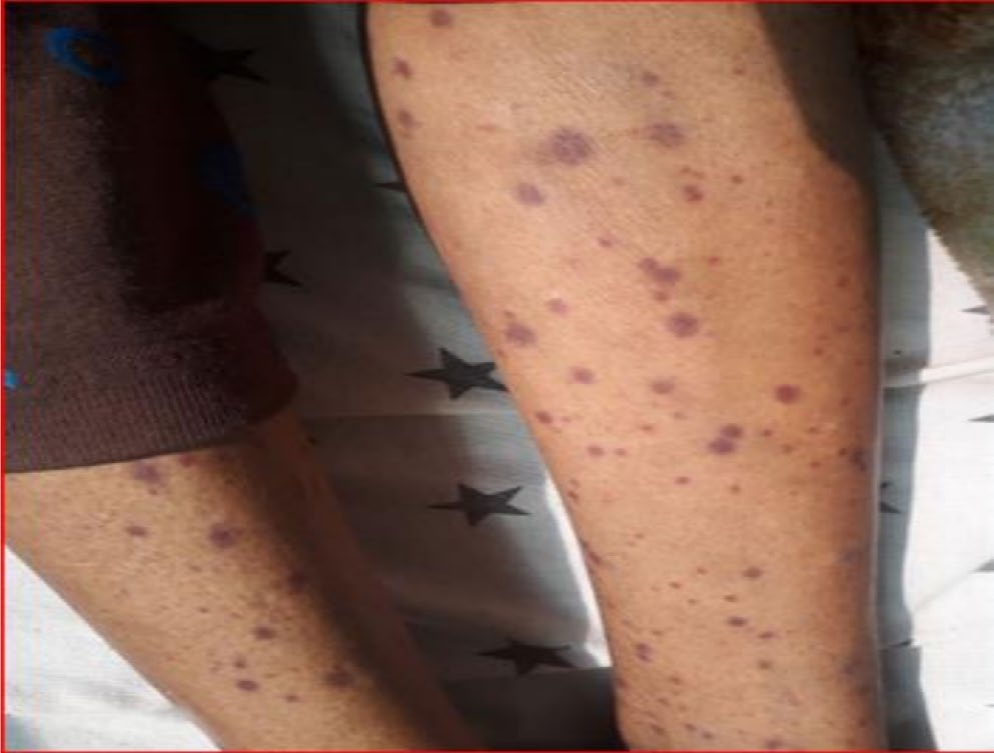
Diffuse petechiae and non‐palpable purpura (lower extremities below knee, admission photo).

On examination, the patient appeared acutely ill and was febrile (38.2°C), tachycardic (104 bpm), and tachypneic (32 breaths/min), with stable blood pressure (130/80 mmHg) and normal oxygen saturation (96% on room air). She was cachectic, with a BMI of 16.8 kg/m^2^, and exhibited conjunctival pallor along with active gum bleeding. There were widespread mucocutaneous petechiae and non‐palpable purpura distributed across her neck, torso, and extremities (Figure 1). Respiratory examination revealed bilateral scattered crepitations, while abdominal and neurological assessments were unremarkable. No palpable lymphadenopathy, hepatosplenomegaly, or signs of jaundice were noted.

## 3 Differential Diagnosis

The diagnostic evaluation encompassed a broad differential for thrombocytopenia in this clinical context. Primary ITP was initially considered given the thrombocytopenia and hemorrhagic manifestations [1, 8], but the presence of systemic symptoms and radiographic findings of miliary TB argued against this diagnosis [14]. Evans syndrome, characterized by concurrent autoimmune hemolytic anemia and ITP [15], was deemed unlikely due to the absence of laboratory evidence of hemolysis and the anemia's resolution with ATT alone. Thrombotic microangiopathies such as thrombotic thrombocytopenic purpura were excluded based on the lack of microangiopathic hemolysis, normal renal function, and coagulation parameters [8].

Hematologic malignancies were carefully evaluated given the constitutional symptoms [16], but the bone marrow examination showing erythroid hyperplasia without blasts or dysplastic changes effectively ruled out marrow infiltration. Connective tissue disorders including systemic lupus erythematosus were considered but deemed improbable given negative autoimmune serologies and absence of multiorgan involvement [17].

Although the combination of constitutional symptoms (fever, weight loss, night sweats) and thrombocytopenia raises a broad differential beyond TB—including Human immunodeficiency virus infection (HIV)‐related cytopenias, opportunistic fungal infections (e.g., chronic histoplasmosis, cryptococcosis) and hematological malignancies such as Non‐Hodgkin lymphoma (NHL)—these alternatives become less likely in our case. First, HIV was excluded by negative serology, and in immunocompetent patients these fungal infections or other opportunistic pathogens are rare and typically have additional features (e.g., lymphadenopathy, hepatosplenomegaly) which were absent [18]. Second, lymphoma often presents with persistent lymphadenopathy, poor or no response to empiric anti‐TB therapy, and may require histological confirmation; in one retrospective cohort, many patients treated empirically for TB later proved to have lymphoma only after biopsy following TB treatment failure [19]. In our patient, clinical improvement (resolution of constitutional symptoms) and normalization of platelet count after anti‐TB therapy strongly argue for TB as the underlying etiology rather than a chronic viral, fungal, or neoplastic process. Finally, although bone‐marrow abnormalities (pancytopenia or thrombocytopenia) have been documented in disseminated TB, these revert with successful therapy. The temporal association between ATT initiation and platelet recovery along with microbiologic confirmation of tuberculosis [20], ultimately established the diagnosis of TB‐associated ITP [21, 22].

## 4 Investigations and Treatment

**FIGURE 2:**
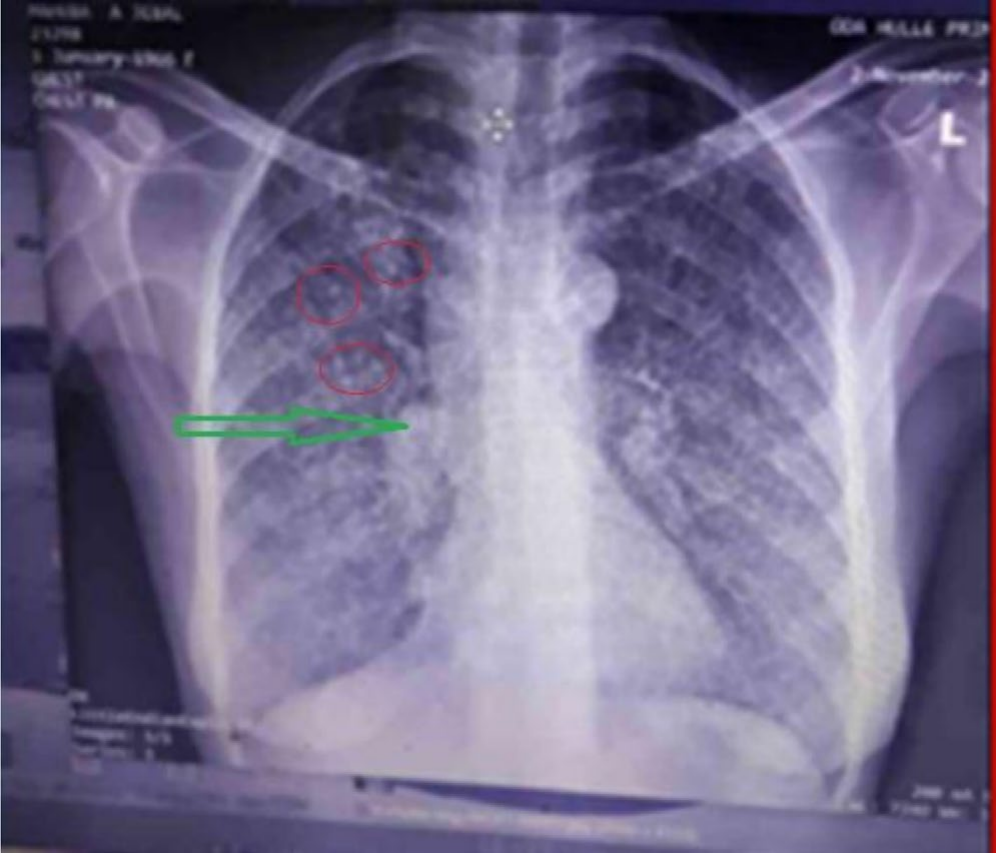
An anteroposterior chest radiograph demonstrates bilateral, diffuse miliary nodules (1–2 mm) and right hilar lymphadenopathy. Red circle indicates miliary nodules and green arrow indicate an enlarged right hilar lymph node, findings characteristic of miliary tuberculosis.

Initial laboratory evaluation revealed severe thrombocytopenia (platelets: 11,000/μL), moderate normocytic‐normochromic anemia (Hb: 8 g/dL), and a normal leukocyte count (5900 cells/mm^3^) (Table 1). The anemia was considered multifactorial, primarily attributed to anemia of chronic inflammation secondary to miliary tuberculosis, compounded by acute blood loss from hemorrhagic manifestations. The peripheral blood smear showed normocytic normochromic red cells and large platelets without schistocytes or blasts. Other common etiologies were investigated and deemed unlikely: the absence of schistocytes on peripheral smear argued against microangiopathic hemolysis; normal lactate dehydrogenase (LDH) and bilirubin levels made significant hemolysis improbable. Bone marrow examination was performed to exclude underlying marrow infiltration or hematologic malignancy given the severity of the cytopenias. Bone marrow aspiration demonstrated erythroid hyperplasia and increased megakaryocytes, consistent with peripheral platelet destruction. Bone marrow smear images were not obtained at that time. Imaging with chest X‐ray revealed a miliary pattern—diffuse, discrete nodular opacities in both lung fields—along with right hilar lymphadenopathy (Figure 2). Sputum analysis confirmed tuberculosis, with positive acid‐fast bacilli (AFB) staining and a GeneXpert‐MTB/RIF assay detecting *Mycobacterium tuberculosis* without rifampicin resistance. The erythrocyte sedimentation rate (ESR) was markedly elevated at 100 mm/h, which corrected to 49.01 mm/h after adjusting for anemia. Laboratory investigations revealed normal renal function tests, coagulation profiles, lactate dehydrogenase (LDH) levels, liver enzymes (including bilirubin), and serum electrolytes (sodium, chloride, potassium, and calcium). Serological testing for VDRL, hepatitis B surface antigen, hepatitis C antibody, *H. pylori* stool antigen, HIV antibody, qualitative antinuclear antibody (ANA), and direct Coombs test were all non‐reactive.

**TABLE 1:** Serial complete blood count parameters of the patient from admission to 1 year of outpatient follow‐up, along with the treatments administered.

CBC variables	Day 1	Day 4	Day 6	2nd month	6th month	1st year	Reference range
WBC count (10^3^/μL)	5.9	10	10	7.2	6.3	5	4–12
RBC count (10^6^/μL)	4.2	4.1	4.2	4.1	4.5	4.9	4.5–5.5
Hematocrit (%)	24.4	24	26.9	27	32.8	33	36.0–46.0
Hemoglobin (g/dL)	8	7.7	8.2	9.1	11	11.3	11–16.5
MCV (fL)	77.5	78	80	79	82	88	82–100
Plt count (10^3^)	11	23	72	149	157	172	150–450
RDW	17.8	17	18	17.3	17	13.2	11.5–14.5
Reticulocyte %	2.2	2.3	2.3	2.2	2.2	1.3	0.7–2.5
Dexamethasone^a^	Yes	Yes	No	No	No	No	—
ATT^b^	No	Yes	Yes	Yes	Yes	No	—

Abbreviations: CBC, complete blood count; MCV, mean corpuscular volume; Plt, platelet; RBC, red blood cell; RDW, red cell distribution width; WBC, white blood cell.

^a^ Dexamethasone 40 mg IV daily for the first 4 days.

^b^ ATT: Antituberculous therapy initiated on fourth day of admission.

Based on the clinical presentation, hematologic findings, and confirmatory microbiological tests, the patient was diagnosed with ITP secondary to miliary tuberculosis. The absence of other underlying causes of thrombocytopenia, along with the temporal association between ATT initiation and platelet recovery, strongly supported this etiology. The subsequent resolution of anemia alongside constitutional symptoms following anti‐tuberculosis therapy further supports the tuberculosis associated inflammatory etiology for anemia.

The patient presented with life‐threatening bleeding due to severe thrombocytopenia, necessitating immediate intervention. High‐dose intravenous dexamethasone (40 mg daily) was initiated to rapidly increase platelet counts and control active hemorrhage. Steroids were discontinued after 4 days once anti‐tuberculosis therapy (ATT) was started, as the underlying miliary TB was considered the primary driver of ITP. The patient was started on the standard Ethiopian TB treatment regimen (2 months of isoniazid, rifampicin, pyrazinamide, and ethambutol, followed by 4 months of isoniazid and rifampicin).

## 5 Outcome and Follow‐Up

**GRAPH 1:**
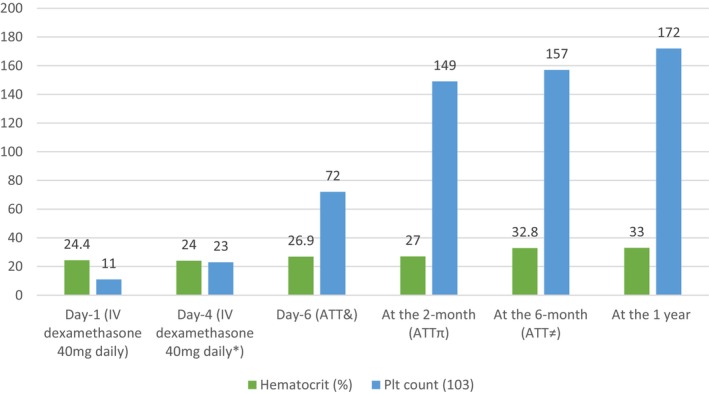
Graphic representation of serial platelet count, hematocrit %, and respective treatment starting from the date of admission and continuing through the first year of follow‐up.

Remarkably, her platelet count showed steady improvement, rising to 72,000/μL by day 6 without further steroid therapy (Table 1 and Graph 1). Following ATT initiation, her constitutional symptoms—including cough, fever, and night sweats—gradually resolved. By the end of the 6‐month ATT course, her hematologic parameters had normalized, with a sustained platelet count of 172,000/μL at 1‐year follow‐up. This complete and durable recovery without additional immunosuppressive therapy strongly supported the diagnosis of TB‐associated ITP. The case illustrates that in TB‐endemic regions, identifying and treating the underlying infection can lead to resolution of secondary ITP, avoiding unnecessary long‐term steroid use.

## 6 Discussion

Although primary ITP is diagnosed by exclusion [8], secondary forms are increasingly linked to infections, autoimmune diseases, and hematologic malignancies [17, 23]. The present case illustrates tuberculosis‐associated ITP, highlighting how miliary tuberculosis may present with severe, life‐threatening thrombocytopenia as its primary hematologic manifestation.

The patient's clinical presentation posed a significant diagnostic challenge, requiring careful differentiation between primary ITP and secondary causes. The presence of constitutional symptoms including fever, night sweats, and weight loss—classic features of tuberculosis [21, 24] provided important clues pointing toward an underlying infectious etiology. This contrasts with typical primary ITP cases, which generally lack systemic manifestations [25]. The rapid platelet recovery following anti‐tuberculosis therapy (ATT), without requiring prolonged immunosuppression, strongly supports the causal relationship between miliary TB and ITP in this case [26, 27]. This observation aligns with existing literature suggesting that infection‐associated ITP often resolves with treatment of the underlying condition [22, 28].

The Ethiopian patient's presentation (miliary TB with severe ITP) resembles several reported cases [5, 6, 11, 29, 30, 31, 32, 33, 34]. For example, Gannepalli et al. described a 42‐year‐old female with miliary pulmonary TB and ITP (platelets ~32,000/μL), whose count normalized within 10 days of ATT and steroids, and Ketema et al. reported an 11‐year‐old male from Ethiopia with miliary TB whose platelet count fully recovered after 6 months of ATT. Like these cases, our patient required anti‐tubercular therapy combined with immunosuppression. In contrast, some cases involved extrapulmonary TB, such as Barbacena et al., who reported lymph node TB with ITP (platelets 3 × 10^9^/L) that improved after IVIG/steroids plus ATT. Across reports, all patients received ATT and most received corticosteroids or IVIG; platelet recovery was achieved in nearly all survivors. Compared to previously reported cases of TB‐associated ITP (TB‐ITP), in which platelet recovery after initiation of anti‐tuberculosis therapy (ATT) typically ranged from 2 days up to 3 months, our patient's rapid and sustained platelet normalization underscores a relatively swift hematologic response to ATT in this context. Differences include patient demographics (our patient is older and female; many reports are younger or male) and TB distribution (some cases had CNS or lymph node TB). Nevertheless, the consistent pattern is that thrombocytopenia in TB usually resolves only after effective ATT, as seen in our case and others (see Table S2).

The management of this case highlights several important therapeutic considerations. While corticosteroids remain first‐line therapy for primary ITP [8], their role in infection‐associated ITP is more nuanced [26]. In our patient, a brief course of high‐dose dexamethasone was employed. The rationale for a short, high‐dose dexamethasone pulse was twofold. Firstly, it provides rapid immunomodulation to increase platelet counts by inhibiting macrophage phagocytosis of antibody‐coated platelets and potentially boosting platelet production, thereby mitigating immediate life‐threatening hemorrhage. Secondly, the brief 4‐day course was judged to carry a minimal risk of exacerbating the TB infection, as the concomitant initiation of effective multi‐drug ATT would immediately begin reducing the mycobacterial burden. In this scenario, the imminent mortality risk from profound thrombocytopenic bleeding outweighed the theoretical risk of transient immunosuppression, which was actively countered by specific anti‐mycobacterial therapy [8, 9, 35]. This approach reflects current understanding that sustained immunosuppression may be detrimental in infection‐driven ITP [27], with treatment of the underlying infection being paramount [21, 28].

The patient's excellent response to ATT alone [20], with sustained platelet recovery at one‐year follow‐up, underscores several key points. First, it reinforces the importance of identifying and treating underlying infections in patients presenting with ITP, particularly in TB‐endemic regions [24]. Second, it demonstrates that prolonged immunosuppression may be unnecessary in cases of infection‐associated ITP [8, 26]. Third, it suggests that bone marrow examination, while helpful in excluding other diagnoses [8], may not be required in all cases when a clear infectious etiology is identified [23].

Tuberculosis remains a major global health challenge, with hematologic manifestations occurring in up to 10%–20% of cases [24, 28]. While anemia is the most common hematologic abnormality in TB [36], thrombocytopenia is relatively rare, with only about 40 well‐documented cases of TB‐associated ITP in the literature [14]. The pathophysiology likely involves multiple mechanisms, including immune complex‐mediated platelet destruction, molecular mimicry between mycobacterial antigens and platelet membrane glycoproteins, and splenic sequestration [21, 22]. Immune complex– or antiplatelet‐antibody–mediated peripheral platelet destruction due to activation of B‐lymphocytes by mycobacterial antigens; impaired platelet production through bone‐marrow involvement; or enhanced splenic sequestration or consumption of platelets in the reticulo‐endothelial system. Given that our patient had disseminated (likely miliary) TB—implying a high mycobacterial antigen load—the immune‐mediated platelet destruction seems most plausible. The prompt and sustained platelet recovery following initiation of anti‐TB therapy (without recurrent thrombocytopenia) further supports a reversible immunologic mechanism rather than irreversible marrow damage or sequestration. Our case contributes to the limited literature and, particularly in Ethiopia where tuberculosis is endemic, offers valuable insights for clinicians practicing in the region [14, 26].

## Author Contributions

**Hayatu Awel Abdela:** conceptualization, data curation, investigation, resources, writing – original draft, writing – review and editing. **Tamirat Godebo Woyimo:** conceptualization, data curation, investigation, resources, writing – review and editing. **Ragasa Getachew Bayisa:** conceptualization, writing – review and editing. **Sisay Tagese Tafese:** conceptualization, writing – review and editing.

## Funding

The authors have nothing to report.

## Ethics Statement

There is no need for institutional authorization to publish the case details.

## Consent

Written informed consent was obtained from the patient before starting the data collection process. The confidentiality and privacy of the patient were assured. Neither the data records nor the extracted data were used for any other purpose. The authors received the necessary written informed consent form. The patient offered written informed agreement to the publication of the case's details and accompanying images.

## Conflicts of Interest

The authors declare no conflicts of interest.

## Supporting information


**Data S1:** ccr371922‐sup‐0001‐supinfo.docx.

## Data Availability

The data supporting the findings of this study are available from the corresponding author upon reasonable request.
