# Supplementary material for: Miliary Tuberculosis With Immune Thrombocytopenia in 50‐Year‐Old Ethiopian Woman: A Case Report and Brief Review of Literature
Source: Clin Case Rep. 2026 Jan 26;14(2):e71922. doi: 10.1002/ccr3.71922 (PMC12834663; doi:10.1002/ccr3.71922)
Supplement: Supplementary file 1 — Data S1: ccr371922‐sup‐0001‐supinfo.docx. [file CCR3-14-e71922-s001.docx]

**Supplementary Table-2:** Literature Review conducted on tuberculosis associated Immune Thrombocytopenia.

| **Reference, Year** | **Patient (Age, Sex, Location)** | **TB Type** | **Platelet Count (at presentation)** | **Diagnostic Methods** | **Treatment (ITP/TB)** | **Outcome** |
| --- | --- | --- | --- | --- | --- | --- |
| **Spedini et al., 2002** | 48 F (African origin, Italy) | Extrapulmonary (mediastinal lymph node) TB | **1×10^9/L** | Chest CT: mediastinal adenopathy; lymph node biopsy (M. tuberculosis culture+) | IV methylprednisolone then 4-drug ATT (HRZE) | Platelet 148×10^9/L after 5 weeks of ATT |
| **Dagaonkar et al., 2012** | 44 F (Mumbai, India) | Disseminated TB (miliary lung + CNS + marrow) | **36,000/mm^3** | HRCT chest: miliary nodules; MRI brain: tuberculomas; bone marrow biopsy (caseating granulomas, normal megakaryocytes) | ATT (initially INH/PZA/EMB + steroid for CNS); IVIG 400 mg/kg×5d; high-dose steroids (prednisone 60 mg) | Platelets 99×10^9/L at 7 mo follow-up; neurological symptoms resolved |
| **Gannepalli et al., 2014** | 42 F (India) | Pulmonary miliary TB | **32,000/µL** | Chest X-ray/HRCT: diffuse fine nodular opacities (miliary pattern); sputum AFB negative | Platelet transfusion; IV dexamethasone; 6 months ATT | Platelet count normalized in 10 days; lung lesions resolved |
| **Ghobrial et al., 2001** | 49 M (USA) | Disseminated TB (pulmonary + nodal) | **Severe ITP** (exact count not stated) | Clinical, radiographic and histologic evidence of disseminated TB; bone marrow biopsy (granulomas) | High-dose IV steroids + IVIG (no response); then ATT | ITP (thrombocytopenia) resolved only after ATT |
| **Malla et al., 2019** | 26 M (India) | Disseminated TB (cervical lymph nodes + Pott’s spine) | **3,000/mm^3** | FNAC of cervical LN: granulomatous lymphadenitis with AFB+; MRI spine: vertebral TB | Platelet transfusion; IVIG 1 g/kg×1; IV dexamethasone (40 mg×4d then taper); ATT (Category I) | Platelets rose to ~150×10^9/L by 2 wk; platelet count 150×10^9/L at 2 weeks, asymptomatic at 10 months |
| **Nasa et al., 2019** | Young M (India) | Extrapulmonary TB (cervical lymph node) | **Severe ITP** (not quantified) | Cervical LN FNA: acid-fast bacilli seen (extrapulmonary TB) | IVIG infusion (1 g/kg) | Platelet count improved with IVIG (bleeding resolved); subsequently treated as TB-related ITP |
| **Barbacena et al., 2024** | 22 F (Portugal, from Guinea-Bissau) | Extrapulmonary TB (cervical lymphadenitis) | **3×10^9/L** | Cervical adenopathy on exam; excisional LN biopsy: caseating granulomas with Langhans cells; IGRA positive | IVIG 1 g/kg×2d; IV methylprednisolone → oral prednisolone; 2 platelet transfusions; ATT (isoniazid, rifampin, ethambutol, pyrazinamide + ofloxacin) | Platelets 164×10^9/L at discharge; bleeding resolved and remained stable on taper |
| **Khan et al., 2020** | 54 M (country not specified) | Disseminated TB | **ITP (asymptomatic)** (count not given) | Workup (imaging, cultures) confirmed disseminated TB | Tapering oral steroids + ATT | Marked and persistent increase in platelet count on steroids + ATT |
| **Ketema et al., 2022** | 11 M (Ethiopia) | Miliary pulmonary TB | **Severe ITP** (not given) | Chest X-ray: diffuse bilateral miliary nodules; TB confirmed by clinical/IGRA or culture | Prednisone + platelet transfusion; 6 months ATT | Platelet count fully normalized after 6 months of ATT |
| **AFB** - Acid-Fast Bacilli, **ATT** - Anti-Tuberculosis Therapy, **CNS** - Central Nervous System, **CT** - Computed Tomography, **d** - Day(s), **EMB** - Ethambutol, F - Female, **FNA / FNAC** - Fine Needle Aspiration (Cytology), **HRCT** - High-Resolution Computed Tomography, **HRZE** - Isoniazid (H), Rifampin (R), Pyrazinamide (Z), Ethambutol (E), **IGRA** - Interferon-Gamma Release Assay, **INH** - Isoniazid, **ITP** - Idiopathic Thrombocytopenic Purpura, **IV** - Intravenous, **IVIG** - Intravenous Immunoglobulin, **L** - Litre, **LN** - Lymph Node, **M** - Male, mo - Month(s), **MRI** - Magnetic Resonance Imaging, **mm³** - Cubic Millimetre, **M. tuberculosis** - Mycobacterium tuberculosis, **PZA** - Pyrazinamide, **TB** - Tuberculosis, **µL** - Microlitre, **wk** - Week(s), **×10⁹/L** - Times 10 to the 9th power per Litre | | | | | | |
